# Contemporary evidence of workplace violence against the primary healthcare workforce worldwide: a systematic review

**DOI:** 10.1186/s12960-023-00868-8

**Published:** 2023-10-13

**Authors:** Hanizah Mohd Yusoff, Hanis Ahmad, Halim Ismail, Naiemy Reffin, David Chan, Faridah Kusnin, Nazaruddin Bahari, Hafiz Baharudin, Azila Aris, Huam Zhe Shen, Maisarah Abdul Rahman

**Affiliations:** 1 Department of Community Health, Faculty of Medicine, Universiti Kebangsaan Malaysia, Cheras, 56000 Kuala Lumpur, Malaysia; 2 The State of Selangor Health Department, Tingkat 9, 10-11, Wisma 16 Sunway Mas, Lot 1, Jalan Persiaran Kayangan, 40100 Shah Alam, Selangor Malaysia; 3 Anaesthesiology Department, Hospital Queen Elizabeth II, Lorong Bersatu Off Jalan Damai, 88300 Kota Kinabalu, Sabah Malaysia

## Abstract

Violence against healthcare workers recently became a growing public health concern and has been intensively investigated, particularly in the tertiary setting. Nevertheless, little is known of workplace violence against healthcare workers in the primary setting. Given the nature of primary healthcare, which delivers essential healthcare services to the community, many primary healthcare workers are vulnerable to violent events. Since the Alma-Ata Declaration of 1978, the number of epidemiological studies on workplace violence against primary healthcare workers has increased globally. Nevertheless, a comprehensive review summarising the significant results from previous studies has not been published. Thus, this systematic review was conducted to collect and analyse recent evidence from previous workplace violence studies in primary healthcare settings. Eligible articles published in 2013–2023 were searched from the Web of Science, Scopus, and PubMed literature databases. Of 23 included studies, 16 were quantitative, four were qualitative, and three were mixed method. The extracted information was analysed and grouped into four main themes: prevalence and typology, predisposing factors, implications, and coping mechanisms or preventive measures. The prevalence of violence ranged from 45.6% to 90%. The most commonly reported form of violence was verbal abuse (46.9–90.3%), while the least commonly reported was sexual assault (2–17%). Most primary healthcare workers were at higher risk of patient- and family-perpetrated violence (Type II). Three sub-themes of predisposing factors were identified: individual factors (victims’ and perpetrators’ characteristics), community or geographical factors, and workplace factors. There were considerable negative consequences of violence on both the victims and organisations. Under-reporting remained the key issue, which was mainly due to the negative perception of the effectiveness of existing workplace policies for managing violence. Workplace violence is a complex issue that indicates a need for more serious consideration of a resolution on par with that in other healthcare settings. Several research gaps and limitations require additional rigorous analytical and interventional research. Information pertaining to violent events must be comprehensively collected to delineate the complete scope of the issue and formulate prevention strategies based on potentially modifiable risk factors to minimise the negative implications caused by workplace violence.

## Introduction

Events where healthcare workers (HCWs) are attacked, threatened, or abused during work-related situations and that present a direct or indirect threat to their security and well-being are referred to as workplace violence (WPV) [1]. Violence in the health sector has increased over the last decade and is a primary global concern [2]. Recent statistical data demonstrated that HCWs were five times more likely to experience violence than workers in other sectors and are involved in 73% of all nonfatal violent work incidents [3]. The experience of WPV is linked to reduced quality of life and negative psychological implications, such as low self-esteem, increased anxiety, and stress [4–6]. WPV is often linked to poor work performance caused by lower job satisfaction, higher absenteeism, and reduced worker retention [7, 8], which may disrupt patient care quality and other healthcare service productivity [9]. Decision-makers and academics worldwide now recognise the seriousness of WPV in the health sector, which has been extensively examined in tertiary settings, particularly emergency and psychiatric departments. Nonetheless, understanding of WPV in primary healthcare (PHC) settings is minimal.

The modern health system has experienced a fundamental shift in delivery systems while moving towards universal health coverage and Sustainable Development Goals (SDGs) [7]. Despite the focus on tertiary-level individual disease management, the healthcare system recently moved towards empowering primary-level patient and community health needs [10]. Robust PHC system delivery provides deinstitutionalised patient care, which includes health promotion, acute disease management, rehabilitation, and palliative services, via primary health units in the community, which are referred to with different terms across countries, such as family health units, family medicine and community centres, and outpatient physician clinics [11–13]. In developing and developed countries, PHC services are associated with improved accessibility, improved health conditions, reduced hospitalisation rates, and fewer emergency department visits [14]. The backbone of this health system delivery is a PHC team of family physicians, physician assistants, nurses, laboratory technicians, pharmacists, social workers, administrative staff, auxiliaries, and community workers [15].

Nevertheless, the nature of PHC service, which delivers essential services to the community, requires direct interaction with patients and family members, thus increasing the likelihood of experiencing violent behaviour [10]. Understaffing occurs mainly due to the lack of comprehensive national data that could offer a complete view of the PHC workforce constitution and distribution, which results in increased responsibilities and compromised patient communication [15]. Considering the current worldwide employment patterns, a shortage of approximately 14.5 million health workers in 2030 is anticipated based on the threshold of human resource needs related to the SDG health targets [16]. Other challenges at the PHC level recently have also been addressed, including long waiting times, dissatisfaction with referral systems, high burnout rates, and limited accessibility in rural areas, which exacerbate existing WPV issues [14].

As PHC system quality relies entirely on its workers, the issue of WPV requires more attention. WPV issues must be examined separately between PHC and other clinical settings to support an effective violence prevention strategy for PHC, given that the violence characteristics and other relevant factors can vary by facility type. In addition, PHC workers also have distinct services, work tasks, and work environments [11]. Since the Alma-Ata Declaration of 1978, interest in conducting empirical studies investigating WPV in the PHC setting has increased worldwide [17]. Nevertheless, a comprehensive systematic review summarising the results from previous studies has never been published. Understanding this issue among workers who serve under a robust PHC system would be equally essential and requires attention to critical dimensions on par with WPV incidents in other clinical settings, especially hospitals. Therefore, this preliminary systematic review of WPV against the PHC workforce analysed and summarised the current information, including the WPV prevalence, predisposing factors, implications, and preventive measures in previous research.

## Methods

### Literature sources

This systematic review was conducted based on the Preferred Reporting Items for Systematic Reviews and Meta-Analyses (PRISMA) 2020 review protocol [18]. A comprehensive database search of the Web of Science, Scopus, and PubMed databases was conducted in February 2023 using key terms related to WPV (“violence”, “harassment”, “abuse”, “conflict”, “confrontation”, and “assault”), workplace setting (“primary healthcare”, “primary care”, “community unit”, “family care”, “general practice”), and victims (“healthcare personnel”, “healthcare provider”, “medical staff”, “healthcare worker”). The keywords were combined using advanced field code searching (TITLE–ABS–KEY), phrase searching, truncation, and the Boolean operators “OR” and “AND”.

### Eligibility criteria

All selected studies were original articles written in English and published within the last 10 years (2013–2023) on optimal sources or current literature. The articles were selected based on the following criteria:

Inclusion criteria

1. Described all violence typology (Types I–IV) and its form (verbal abuse, physical assault, physical threat, racism, bullying, or sexual assault);
2. The topic of interest concerned every category of PHC personnel (family doctor, general practitioner, nurse, pharmacist, administrative staff).

Exclusion criteria

1. The violence occurred in a tertiary or secondary setting (during training/industrial attachment at a hospital);
2. Case reports or series, and technical notes.

### Study selection and data extraction

**Fig. 1:**
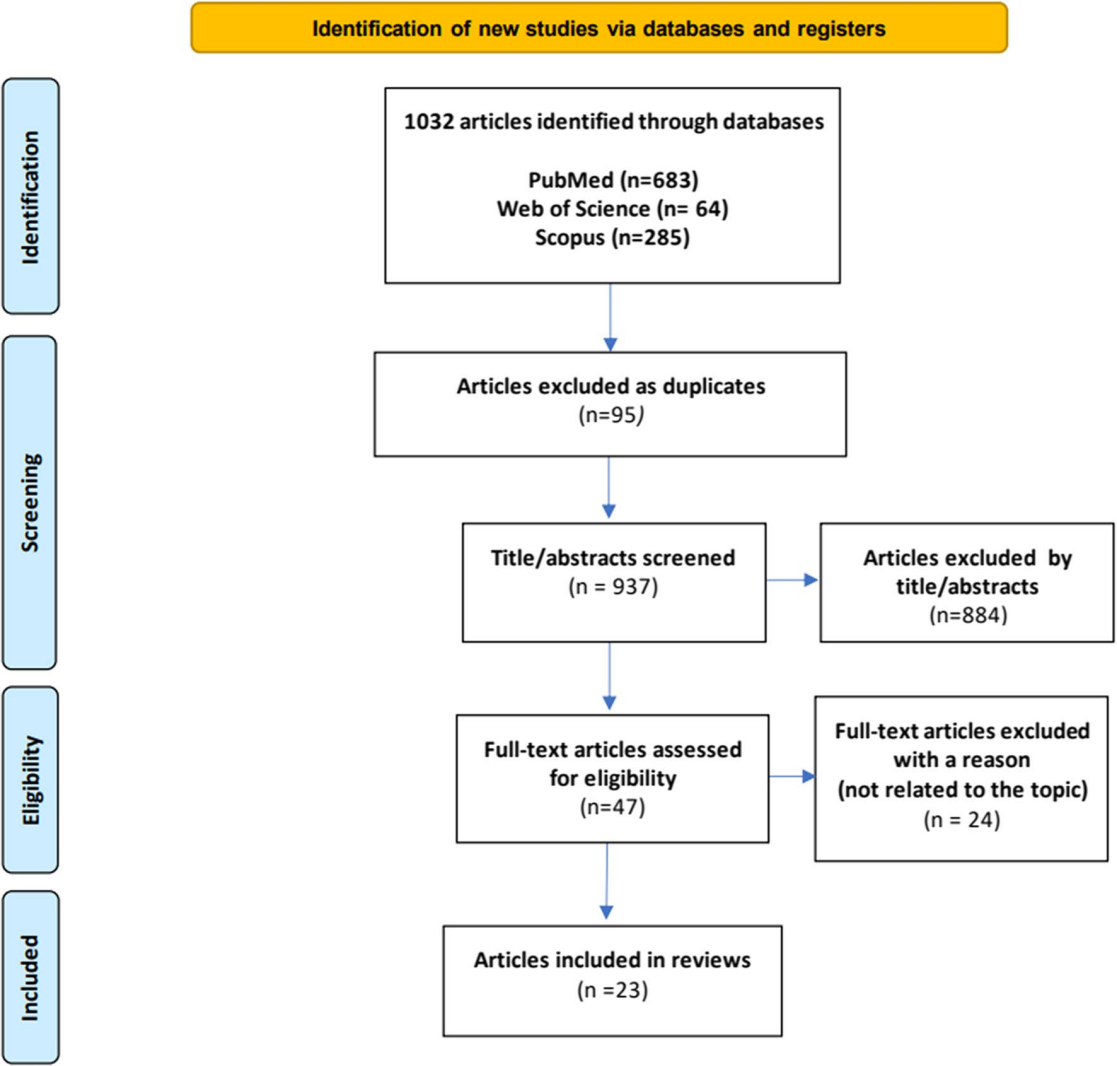
PRISMA flow diagram

All research team members were involved in screening the titles and abstracts of all articles according to the inclusion and exclusion criteria. All potentially eligible articles were retained to evaluate the full text, which was conducted interchangeably by two teams of four members. Differences in opinion were resolved with the research team leader’s input. Before the data extraction and analysis, the methodological quality of the finalised article was assessed using the Mixed-Methods Appraisal Tool (MMAT). Based on the outcomes of interest, the information obtained from the included articles was compiled in Excel and grouped into the following categories: (i) prevalence, typology, and form of violence, (ii) predisposing factors, (iii) implications, and (iv) preventive measures. Figure 1 depicts the article selection process flow.

## Results

### General characteristics of the studies

Forty-three articles were potentially eligible for further consideration, but only 23 articles provided information that answered the research questions (Table 1) [13, 19–40]. The studies mainly covered 16 countries across Asia, Europe, and North and South America, thus providing good ethnic or cultural background diversity. All included articles were observational studies. Sixteen studies were quantitative descriptive studies conducted through self-administered surveys using different validated local versions of WPV study tools (response rate: 59–94.47%). Four qualitative studies collected data through in-depth interviews and focus group discussions. The remaining studies were mixed-method studies that combined quantitative and qualitative research elements. Of the 23 studies, 15 involved various categories of healthcare personnel, seven involved primary clinicians, and one involved pharmacist.

**Table 1:** General characteristic of studies

No	Title	Author (year)	Study design	Study Location	Study Population (Total Sample)	Survey method	Survey instrument
1..	Workplace violence against medical staff in healthcare facilities in Barbados [19]	Abed et al. (2016)	Cross-sectional	Barbados	141 Respondents: Nurses, doctors (72% response rate)	Self-administered survey	Modified version of the standard WHO WPV questionnaire
2.	Aggression towards the GP: Can we profile the GP-victim? A cross-sectional survey among GPs [20]	Demeur et al. (2018)	Cross-sectional	Belgium	246 General practitioners (GPs) (67% response rate)	Online survey	Validated Likert scale questionnaire consisting of socio-demographic background details, questions related to violence and harassment (developed based on a literature review) and the “Big Five personality traits” questionnaire
3.	Violence in general practice: A gendered risk? [21]	Elston and Gabe (2016)	Mixed method	England	697 GPs (62% response rate) 32 GPs	Self-administered survey In-depth interview, focus group discussion	Data were mainly drawn from a UK Economic and Social Research Council (ESRC)-funded study of violence against professionals in the community Semi-structured questionnaire developed based on a literature review of previous research and policy documents and following feminist researchers’ critique of conventional criminology, querying the characteristics of violence, the context for the most recent incidents of violence, practice organisation and environment, and GPs. Biographical backgrounds were also collected, and how they managed the risk of violence and attempted to minimise possible harm
4.	Prevalence and risk factors associated with workplace violence against general practitioners in Hubei, China [22]	Gan et al. (2018)	Cross-sectional	China	1015 GPs (86% response rate)	Self-administered survey	Chinese version of the Workplace Violence Scale developed by Wang et al. 2006, which consists of five sub-sections related to social background and work-related factors
5.	Violence towards personnel in out-of-hours primary care: A cross-sectional study [23]	Joa and Morken (2012)	Cross-sectional	Norway	536 Respondents: Physicians, nurses, others (75% response rate)	Self-administered survey	Australian questionnaire on occupational violence among GPs that consists of a few sections of the core study domains, such as individual work-related factors
6.	Primary care clinician and clinic director experiences of professional bias, harassment, and discrimination in an underserved agricultural region of California [24]	Ko and Dorri (2019)	Qualitative	USA	26 Respondents: Primary care clinicians and clinic directors	In-depth interview	Interview session guided by an open-ended questionnaire on challenges, strategies, and personal journeys during practice
7.	Users’ perception of violence and conflicts with professionals in primary care centers before and during COVID-19. A qualitative study [25]	Pina et al. (2022)	Qualitative	Spain	80 Respondents: Primary care services users	Focus group discussion	Semi-structured questionnaire to evaluate conflicts between users and professionals in primary care (regarding organisational aspects, HCWs’ weaknesses, and users’ attitudes/behaviours) during COVID-19
8.	Sources of conflict and prevention proposals in user violence towards primary care staff: A qualitative study of the perception of professionals [26]	Pina et al. (2022)	Qualitative	Spain	44 Respondents: HCW of primary care services (miscellaneous)	Focus group discussion	Semi-structured questionnaire to evaluate conflicts between users and professionals in primary care (regarding organisational aspects, HCWs’ weaknesses, and users' attitudes/behaviours) during COVID-19
9.	Workplace violence types in family health, offenders, reactions, and problems experienced [27]	Sturbelle et al. (2020)	Mixed method	Brazil	Phase 1: 106 Respondents: Miscellaneous (response rate: not available [NA]) Phase 2: 18 Victims	Phase 1: In-depth interview Phase 2: Self-administered survey	Phase 1 (Quantitative) Modified version of the standard WHO WPV questionnaire Phase 2 (Qualitative) Semi-structured open-ended questionnaire querying potential WPV among professionals who work in the Family Health Unit (FHU) that determined that the work conditions and organisation influence these problems
10.	Aggression and violence against primary care physicians—A nationwide questionnaire survey [28]	Vorderwülbecke et al. (2015)	Cross-sectional	Germany	831 GPs (59% response rate)	Self-administered survey	Validated questionnaire adapted from original questionnaires (international sources)
11.	Organizational safety climate and workplace violence among primary healthcare workers in Malaysia [29]	Rajakrishnan et al. (2022)	Cross-sectional	Malaysia	838 Respondents: Miscellaneous (83% response rate)	Self-administered survey	Validated questionnaire consisting of two main sections: WPV (WHO–ILO) and organisational safety climate (OSC, NOSACQ-50) to elicit information on OSC
12.	Violence against health workers in family medicine centers [30]	Al-Turki et al. (2016)	Cross-sectional	Saudi Arabia	270 Respondents: Miscellaneous (response rate: NA)	Self-administered survey	Modified version of the standard WHO WPV questionnaire
13.	Violence against healthcare workers at primary care centers in Dammam and Al Khobar, Eastern Province, Saudi Arabia, 2019 [13]	Alsmael et al. (2020)	Cross-sectional	Saudi Arabia	360 Respondents: Miscellaneous (64% response rate)	Self-administered survey	Modified version of the standard WHO WPV questionnaire
14.	The impact of patient aggression on community pharmacists: A critical incident study [31]	Irwin et al. (2013)	Qualitative	Scotland	18 Pharmacists	In-depth interview	Semi-structured open-ended questionnaire with three main focuses: recount memory of incidents encountered, query of the general causes of patient aggression, and the supportive actions and use of preventive measures/damage controls
15.	Frequency and forms of workplace violence in primary health care [32]	Jatic et al. (2019)	Cross-sectional	Bosnia	558 Respondents: Medical doctors, nurses (57% response rate)	Self-administered survey	Validated questionnaire consisting of seven topics: socio-demographics, individual working characteristics, type of violence, consequences of violence, mental disorders associated with WPV, reaction to the violence, and education on preventive measures
16.	How the medical culture contributes to coworker-perpetrated harassment and abuse of family physicians [33]	Miedema et al. (2012)	Mixed method	Canada	Phase 1: 3802 GPs (response rate: NA) Phase 2: 41 GPs	Self-administered survey Telephone and in-person interviews	Survey package consisting of a questionnaire and a card with a request for an interview with participants Semi-structured open-ended questionnaire querying the challenges during practice
17.	Aggressions towards primary health care workers in Madrid, Spain, 2011–2012 [34]	Toro et al. (2015)	Cross-sectional	Spain	11,525 Respondents: Miscellaneous	Secondary data	Study data were extracted from the notification survey system used by the Madrid Health System to report events of violence or aggression at primary care centres
18.	User violence and psychological well-being in primary health-care professionals [35]	Cecilia et al. (2017)	Cross-sectional	Spain	574 Respondents: Doctors, nursing staff, non-health staff (response rate: NA)	Self-administered survey	Validated questionnaire consisting of the following sections: Healthcare Workers’ Aggressive Behaviour Scale-Users–Primary Healthcare Version (HABS–U-PHC), Goldberg’s General Health Questionnaire, overall job satisfaction (OJS), and the Jefferson Scale of Physician Empathy (JSPE)
19.	Encouraging employees to report verbal violence in primary health care in Serbia: A cross-sectional study [36]	Marina et al. (2017) 2017	Cross-sectional	Serbia	1526 Respondents: Physicians, nurses (86.8% response rate)	Self-administered survey	Modified version of the standard WHO WPV questionnaire
20.	Does workplace violence exist in primary health care? Evidence from Serbia [37]	Marina et al. (2015)	Cross-sectional	Serbia	1526 Respondents: Physicians, nurses (87% response rate)	Self-administered survey	Modified version of the standard WHO WPV questionnaire
21.	Assessment of non-physical user violence and burnout in primary health care professionals. The modulating role of job satisfaction [38]	David Pina et al. (2022)	Cross-sectional	Spain	574 Respondents: Medical staff, nursing staff, support personnel (80.60% response rate)	Self-administered survey	Validated questionnaire consisting of four sections: Socio-demographic details, HABS–U-PHC, OJS, and the Maslach Burnout Inventory-General Survey (MSI-GS)
22.	Prevalence and associated factors for workplace violence among general practitioners in China: A national cross-sectional study [39]	Feng et al. (2022)	Cross-sectional	China	4376 GPs (95% response rate)	Self-administered survey	Chinese version of the Workplace Violence Scale developed by Wang et al. 2006
23.	Physician–nurse conflict resolution styles in primary health care [40]	Delak and Širok (2018)	Cross-sectional	Slovenia	298 Respondents: 173 nurses, 125 physicians (49% response rate)	Self-administered survey	Thomas–Kilmann Conflict Mode Instrument

### Prevalence, typology, and form of violence

14 studies focused on the prevalence of patient- or family-perpetrated violence (Type II), three studies focused on co-worker-perpetrated violence (Type III), while six studies reported on both type II and III violence (Table 2). Evidence of domestic- and crime-type violence (Types I and IV) was not found in the literature. In most studies, the primary outcome was determined based on recall incidents over the previous 12 months. The reported prevalence of violence against was 45.6–90%. The incidence rate of verbal abuse was 46.9–90.3%, which rendered it the most commonly identified form of violence, followed by threats or assault (13–44%), bullying (19–27%), physical assault (15.9–20.6%), and sexual harassment (2–17%). The reported prevalence of violence against doctors was 14.0–73.0%, followed by that against nurses (6.0–48.5%), pharmacists (61.8%), and others (from 40% to < 5%). Patients and their families were the main perpetrators of violence, followed by co-workers or supervisors (Table 2).

**Table 2:** The prevalence, typology, and form of WPV against primary healthcare workers

No	Author (year)	Typology of Violence	Duration of WPV	Average Rate of WPV	Rate of WPV Experienced (by Profession)	Perpetrators	Form of Violence
1.	Abed et al. (2016)	II and III	12 months	63%	–	Patients: 64% Co-workers: 21%	Verbal abuse: 60% Bullying: 19% Sexual harassment: 7% Physical violence: 3% Racial harassment: 3%
2.	Demeur et al. (2018)	II	12 months Monthly	79.8% 11.3%	–	Known patients: 55.1% Psychiatric patients: 32.1% Patients under the influence of alcohol/drugs: 11.7%	Verbal aggression: 89.8% Psychological aggression: 21.1% Physical aggression: 20.6% Sexual aggression: 7.7%
3.	Elston and Gabe (2016)	II	Lifetime	78%	–	Main perpetrators: Clients of Primary Healthcare service	Male: Physical violence: 13% Threats: 13% Verbal violence: 74% Female: Physical violence: 7% Threats: 18% Verbal violence: 8%
4.	Gan et al. (2018)	II and III	12 months	62.2%	–	–	Physical violence: 18.9% Non-physical violence: 61.4% Verbal abuse: 54.4% Threats: 33.8% Sexual harassment: 22.7% Physical assault: 18.9% Sexual assault: 7.6%
5.	Joa and Morken (2012)	II	12 months	–	–	Main perpetrators: Patients under the influence of drugs/alcohol or with severe mental illness	Verbal abuse: 78% Threats: 44% Physical abuse: 13% Sexual harassment: 9%
6.	Ko and Dorri (2019)	III	Lifetime	–	–	Main perpetrators: Colleagues and administrators	Inter-profession bias, harassment, and discrimination
7.	Pina et al. (2022)	II	–	–	–	Main perpetrators: Aggressive and demanding patients	–
8.	Pina et al. (2022)	II	–	–	–	Main perpetrators: Aggressive and demanding patients	–
9.	Sturbelle et al. (2019)	II and III	12 months	69.8%	Clinic Health Assistance: 33% Technician: 21% Nurse: 14% Doctor: 6%	Users: 71.6% Family/companions: 6.4% Colleagues: 11% Leaders: 9.2%	–
10.	Vorderwülbecke et al. (2015)	II	Lifetime 12 months	91% 73%	–	Patients: 76% Patients’ relatives: 17% Patients under the influence of alcohol/drugs or with mental illness: 63%	Verbal abuse: 79% Intimidation: 38% Physical violence: 16% Sexual abuse: 17% Verbal abuse: 54% Intimidation: 21% Physical violence: 7% Sexual abuse: 10%
11.	Rajakrishnan et al. (2022)	II	12 months	68.5%	Nurse: 44.8% Doctor: 15.1% Officer: 7.3% Technical: 5.9% Support staff: 5.2% Other: < 5.0%	Main perpetrators: Patients/visitors, followed by colleagues, superiors/management, the public, and others	Verbal abuse: 65% Bullying: 27% Physical violence: 6% Sexual harassment: 2%
12.	Al-Turki et al. (2016)	II and III	12 months	45.6%	Clerk: 68.4% Pharmacist: 61.9% Doctor: 44.4% Technician: 40.0% Nurse: 36.0%	Patients: 71.5% Others (companions or co-workers): 28.5%	Physical violence: 6.5% Non-physical violence: 99.2%, including verbal violence (94.3%) and intimidation (22.0%)
13.	Alsmael et al. (2020)	II	12 months	46.9%	–	Patients: 74% Others (companions or co-workers): 26%	Verbal violence: 90% Intimidation: 34.3% Physical violence: 3%
14.	Irwin et al. (2013)	II	Daily Weekly Monthly		–	Main perpetrators: Patients and family members	Verbal aggression: n = 26 Physical aggression: n = 18 Incidents beginning with verbal aggression and ending with physical aggression: n = 7 Physical aggression directed at an inanimate object: n = 16
15.	Jatic et al. (2019)	II	12 months	90.3%	–	Main perpetrators: Patients and family members	Verbal violence: 89.2% Indirect physical violence: 74.7%
16.	Miedema et al. (2012)	II	Lifetime	–	–	–	–
17.	Toro et al. (2015)	II	12 months	–	Family doctor: 48.48% Nurse: 17.63% Auxiliary nurse: 1.29% Assistant: 24.37% Support staff: 1.72% Midwife: 0.43% Physiotherapist: 0.17% Social worker: 0.17% Unknown: 0.77%	Patients: 67.8% Unknown: 3% Companions: 28.1%	Physical violence: 4.7% Threats: 52.8% Coercion: 25.7% Insults: 75.2% Material damage: 31.2%
18.	Cecilia et al. (2017)	II and III	12 months	–	–	Patients/co-workers	–
19.	Marina et al. (2017)	II	12 months	48%	Physician: 28.5% Nurse: 63.4% Other: 8.1%	Clients: 52.1% Relatives: 14.3% Co-workers: 21.4% Management: 10.8% General public: 0.5%	Verbal violence: 48%
20.	Marina et al. (2017)	II	12 months	2.6%	Physician: 28.6% Nurse: 62.1% Other: 9.2%	–	Physical violence: 1.9% Verbal abuse: 43.5% Mobbing: 5.7% Sexual harassment: 0.4% Racial harassment only: 0.2%
21.	Pina et al. (2022)	III	12 months	–	–	–	–
22.	Feng et al. (2022)	II and III	12 months	14.26%	–	Patients: 50.64% Patients’ families: 40.38% Colleagues/supervisors: 0.16%–1.28% Public: 3.21%	Verbal abuse: 13.44% Threats: 9.23% Verbal sexual harassment: 4.68% Physical assault: 4.59% Physical sexual assault: 2.29%
23.	Delak and Širok (2018)	III	–	–	–	–	–

### Predisposing factors of WPV

- Victims’ personal characteristics

Several socio-demographic factors were identified as predictors of WPV. Male gender and female gender were associated with risk of physical violence [21–23] and non-physical violence [12, 19, 24, 32, 35, 39], respectively. Nevertheless, a specific form of non-physical violence, such as coercion, was also reported less frequently among women [34]. A minority group of HCWs with individual sexual identities perceived a severe form of intra-profession violence, such as threats to their licenses [24]. Being young presented a higher risk for violence, especially sexual harassment, and was frequently complicated by physical injury [23, 27, 34]. A personality trait study demonstrated a significant association between aggression incidents with “reserved” and “careless” personality types [20]. Regarding professional background, medical workers were more vulnerable to physical violence compared to non-medical workers [12, 22, 34]. Nurses faced a higher risk of WPV than others [19, 23, 27, 37]. Nevertheless, non-medical staff were also vulnerable to physical violence [35]. Due to less work experience, certain HCWs were identified as vulnerable to violence [22, 26, 35]. Furthermore, violent clinic incidents could occur due to poor professional–client relationships triggered by workers’ attitudes, such as a lack of communication and problem-solving skills [25, 26] (Table 3).

- Perpetrators’ personal characteristics

**Table 3:** Predisposing factors, impact, and coping mechanisms regarding WPV among primary healthcare workers

No	Author (year)	Predisposing factors	Implications	Preventive measures/coping mechanisms
1.	Abed et al. (2016**)**	Victims’ characteristic: Female nurses were more predisposed to experience violent incidents than male physicians	–	–
2.	Demeur et al. (2018)	Victims’ characteristics (personality traits): GPs with “reserved” and “careless” personality types were likelier to experience aggression GPs with “innovative”, “challenging”, or “confident” personality types were also at increased risk, but to a lesser extent than those with “reserved” and “careless” personalities GPs with “efficient” and “innovative” personalities were likelier to report incidents Male GPs and those with “efficient” personalities felt safer. GPs with “confident” and “cautious” personalities were likelier to feel unsafe	–	–
3.	Elston and Gabe (2016)	Victims’ characteristics: Physical assaults and threats were much rarer and more likely to be reported by men Female GPs’ lower risk of threats and assaults might partly reflect their greater likelihood of adopting specific personal risk reduction measures than their male colleagues	Implications of violence: A few assault cases that resulted in physical injury Female GPs were significantly more likely to report feeling afraid of becoming a victim of violence in the future Specific incidents were mostly rated as having no lasting effect on GPs’ mental or physical health or professional practice A few older male doctors took early retirement due to being assaulted	Safety measures: Female doctors consistently adopted the following safety measures: Checking patient notes in advance Leaving the door ajar when seeing certain patients Accompanied when seeing certain patients Accompanied on visits to certain patients Leaving visit schedule with a colleague Carrying a personal alarm Attending self-defence course
4.	Gan et al. (2018)	Victims’ characteristics: Physical violence was positively associated with: Male GPs Higher professional level Non-physical violence was positively associated with: Less-experienced GPs Those with administrative responsibility Work-related factors: Physical violence was positively associated with: Lower average monthly income	–	–
5.	Joa and Morken (2012)	Victims’ characteristics: Significantly more nurses were associated with verbal abuse Males had a higher risk for physical abuse Higher age was associated with a lower risk for sexual harassment	–	–
6.	Ko and Dorri (2019)	Victims’ characteristics: A minority group who self-identified as female, non-white, and with a certain sexual orientation experienced WPV in their professional relationships with colleagues and health care staff Discriminatory acts against members of sexual and gender minority groups were the most severe, and included threats to licensure and denial of hospital admitting privileges	Post-violence events: Minority groups described burnout from bias, harassment, and hostility in their professional relationships with colleagues and healthcare staff Harassment and institutional discrimination were factors in respondents’ decisions to change practices or leave the region entirely	–
7.	Pina et al. (2022)	Victims’ characteristics: Inappropriate HCW attitude, lower motivation, lack of communication skills, problem-solving deficits Administrative staff lacked communication skills, assertiveness, or empathy Post-COVID-19 adaptation: Users noted that medical staff were perceived as distant and occasionally did not provide sufficient information on users’ health status Perpetrators’ characteristics: Primary health care users’ inappropriate attitude; demanding users, disrespecting rules, apathetic users, language barrier Work-related factors: Organisational deficit: Management of medical appointments by telephone, with users reporting great difficulties in being attended. Online appointments were not a suitable alternative for all users, as it was difficult for low-income and elderly users to use this method Management of emergencies in primary care: Triaging was conducted by non-health administration staff in a low-privacy or less confidential context Uncertain waiting time for consultations: Users reported not seeing the doctor or not being informed of the additional waiting time despite having an appointment at a specific hour. A variant of this also occurred for telematic consultations, where it was uncertain when the call would be received	–	–
8.	Pina et al. (2022)	Work-related factors: Professionals perceived a lack of training or education in themselves, causing violence Absence of functional multidisciplinary teams; deficits in coordination and communication between professionals. A user might receive different, occasionally contradictory messages from different professionals. This could be annoying and confusing, causing violent situations The time allocated to each consultation could be unrealistic, causing overload, poor attendance, and care delays, ultimately causing user conflicts Victims’ characteristic: WPV was due to depersonalised or dehumanised treatment by some professionals, which resulted in communication and empathy deficits Perpetrators’ characteristics: Poor patient–professional relationship induced violence during certain points as follows: Inappropriate use of primary care, especially use of the emergency system. Some users claimed that their consultation was serious with the intention of avoiding making an appointment and waiting to be seen Some users used the service to avoid facing external conflict. For example, using sick leave for malingering	–	–
9.	Sturbelle et al. (2019)	Victims’ characteristic: WPV victims were mainly younger workers and nursing staff Social factor: Locations in areas of trafficking were related to professionals’ exposure to violence Work-related factors: The reception unit was at the greatest risk of aggression compared to other units as it is an environment that requires staff to listen to users’ chief complaints, and the first point to determine the patient’s treatment flow The lack of human and material resources generated exhaustion among professionals and user dissatisfaction, causing poor patient–professional relationships and inducing WPV	Implications of violence: Violence caused damage that influenced work productivity and quality This resulted in dissatisfied professionals who did not feel recognised and had fragile emotions, and decided to leave their job Conflicted relationships with colleagues, bosses, and users	–
10.	Vorderwülbecke et al. (2015)	Social factor: Financially weaker practice clientele were associated with WPV	–	–
11.	Rajakrishnan et al. (2022)	Social factor: WPV prevalence in the past 12 months was highest among HCWs working in larger districts (77.7%) compared to smaller districts (22.3%). Large districts: district health offices with > 500 HCW and serving a population of ≥ 500,000 people Work-related factor: Low level of organisational safety climate was significantly associated with WPV	–	–
12.	Al-Turki et al. (2016)	Work-related factors WPV was significantly associated with working multiple shifts, and evening or night shifts WPV was significantly associated with the lack of an supportive environment to report violence	Implications of WPV: No change: 56.6% Reduced work performance: 31.1% Feeling shame or guilt: 4.9% Feeling sadness or stress: 2.5% Other consequences: 4.9%	Post-violence events: Coping mechanism: 48.0% of HCWs who experienced violence did nothing, 38.2% actively reported it to their supervisors (30.9%), the police (4.9%), 14.5% passively reacted by consulting a colleague or friend (13.8%) or discussing the violence with the offender and resolving the conflict (0.8%) Reasons for underutilising reporting systems: belief that reporting was not an efficient reaction (69.4%), fear of losing their job (12.5%), unknown reasons (11.1%), other causes (6.9%) Most HCWs who experienced violence were either unsatisfied (45.9%) or very unsatisfied (25.4%) with how the violent event was managed
13.	Alsmael et al. (2020)	NA	Implications of WPV: None: 73.8% Decreased work performance: 17.3% Feeling punished: 2.4% Feeling shame or guilt: 1.8% Absenteeism: 1.2% Injury (did not need medical care): 0.6% Injury (needed medical care): 0.6% Other: 13.1%	Post-violence event: Reaction to the event: none (46.7%), reported to supervisor (46.2%), requested to move from workplace (4.7%), consulted colleague or friend (5.9%), reported to police (5.9%), other (4.7%) Reason for not reporting: fear of revenge (1.2%), fear of losing job (3.0%), feeling ashamed/guilty (3.0%), not efficacious (39.1%), do not know (21.4%)
14.	Irwin et al. (2013)	Work-related factor: Uncertain waiting times were the main reason for WPV Perpetrators’ characteristics: Lack of understanding of the pharmacist’s role in supplying medication was a main reason for WPV Patients became aggressive when answering questions related to their medication	Impacts of violence: Physiological effects: Aggressive incidents remained in victims’ thoughts persistently after the event, while a few developed anxiety Cognitive impact: Potential risk of dispensing error or near miss occurred. Others reported reduced concentration or requiring a “time-out” after the incident due to inability to focus Emotional effect: Emotional distress (upset and crying), followed by mild distress (discomfort), witnessing emotional distress in other staff members, self-directed anger Social impact: Hesitancy to engage with patients, deciding to change careers, concerned about patients after the event	During violence event: Pharmacists described using non-technical skills in response to aggressive behaviour: Leadership: most interviewees felt that part of their job role was to take the lead in any aggressive interaction, protect junior members, and take control of the situation Task management: The pharmacists considered management of an aggressive incident to include three key factors: Management of pharmacy staff, management of the aggressive patient, and prescription processing Situational awareness: Maintaining clear exits and positioning staff next to the telephone were vital to maintain pharmacists’ and staff members’ safety. Respondents needed to monitor other staff members’ actions during an incident to ensure that they could maintain a clear idea of what the staff would be able and unable to do if the incident escalated Decision-making: Refusing to interact with the aggressive patient further by asking them to leave the shop premises. However, several pharmacists knew that refusing medication due to aggression might provoke the patient further
15.	Jatic et al. (2019)	Victims’ characteristic: Female gender was significantly associated with verbal violence Workplace factor: Workplace setting (urban) was significantly associated with indirect physical violence	–	–
16.	Miedema et al. (2012)	Work-related factors: Abusive behaviour was regularly modelled in the workplace, which contributed to abuse being perpetuated across generations. One respondent reported that abusive behaviour started in medical school Professional hierarchy discrimination within the medical community, in which specialists are highly valued and family physicians less valued Different pay schedules and scales were important factors in perpetuating professional hierarchies Shortage of physicians was also an important factor contributing to abusive experiences within the medical system Lack of policy and follow–up procedure were not mentioned	–	–
17.	Toro et al. (2015)	Victims’ characteristics: Coercion was less frequent when the worker was female Women more frequently inflicted insults Workers aged < 30 years were at greater risk of material damage at consultation, which increased further for medical staff Perpetrators’ characteristics: Physical assault by patients was almost three times more frequent than that by accompanying persons WPV was frequent when the aggressor was between 51 and 60 years Aggressors aged between 19 and 60 years coerced workers 2–3 times more than those aged > 60 years Work-related factors: Non-medical staff were at lower risk of being physically assaulted than medical staff Medical staff experienced more than twice the risk of experiencing coercion than non-medical staff	Impacts of violence on professionals: No consequences: 90.4% Psychological impact: 5.8% Injuries: 3.6% Work leave: 0.9%	Post-assault intervention: Letter: 50.2% Organisational measures in health centre: 11.2% Change of professional requested by assaulted worker: 18.2% Change of health centre: 0.9% Change of doctor or nurse by patient’s choice: 20.3% Other: 11.6%
18.	Cecilia et al. (2017)	Victims’ characteristic: Women reported more exposure to non-physical violence than men Work-related factors: Professionals with fewer years of professional experience presented higher scores for non-physical violence Professionals who did not receive continued training presented higher scores for non-physical violence Non-health staff were prominent among the professions most exposed to non-physical user violence, followed by doctors, and finally nursing staff	Impact of violence on professionals’ well-being: Psychological well-being: Higher General Health Questionnaire (GHQ) score (indicating poor psychological well-being) among HCWs who experienced WPV Job satisfaction: Higher GHQ score among HCWs dissatisfied with aspects related to their work Empathy: Higher scores in empathy factors were related to lower scores on the total GHQ scale (indicating better psychological well-being). Greater empathy prevents psychological distress in primary care professionals	–
19.	Marina et al. (2017)	Work-related factors: WPV was positively associated with: Interaction with patients between 6 PM and 7 AM Interaction with patients during work	–	Coping mechanism post-violence event: Most respondents (70.1%) did not take any action following WPV Source of action taken: management (67.7%), employer (24.0%), union (0%), association (0%), police (4.2%) Most respondents (44.6%) were highly dissatisfied with the manner in which the incident was handled Main reason for not reporting the incident: it was not important (14.8%), feeling ashamed (2.5%), feeling guilty (0%), afraid of negative consequences (19.2%), feeling useless (74.9%), did not know to whom to report (15%)
20.	Marina et al. (2015)	Work-related factors: WPV was positively associated with: Interaction with patients between 6 PM and 7 AM Nurses as a professional group Working with preschool children WPV was negatively associated with: Encouragement to report violence Number of staff in the same work setting (> 20 staff)	–	–
21.	Pina et al. (2022)	Work-related factor: Non-physical violence and low intrinsic and extrinsic job satisfaction modulated non-physical violence, cynicism, and emotional exhaustion	–	–
22.	Feng et al. (2022)	Victims’ characteristic: Female GPs were less likely to encounter WPV Social factor: GPs practising in rural areas were less likely to encounter WPV Work-related factors: Less likely to encounter WPV: Made occasional home visits Worked in a fair or good practice or work environment Had a fair or good relationship with patients GPs who served > 20 patients per day and worked overtime occasionally	–	Coping mechanism post-violence event: No action taken: 31.92% Tried to pretend it never happened: 14.82% Stopped the perpetrators: 29.80% Told friends/family: 9.28% Told colleagues: 24.92% Sought help from managers: 33.55% Sought help from union: 5.54% Called the police: 17.79% Transferred to another position: 0.80% Completed a WPV report: 11.38% Prosecuted: 0.64%
23.	Delak and Širok (2018)	–	–	Physician–nurse conflict resolution style: The most predominant conflict resolution styles were compromising (44.3%) and avoiding (42.3%) The next most predominant conflict resolution styles were accommodating (7.7%), collaborating (3.4%), and competing (2.3%) The nurses’ and physicians’ predominant conflict resolution styles were avoiding and compromising, respectively, but there were no statistically significant differences Conflict resolution style were statistically significantly different according to gender, education, and tenure Men mainly chose compromising (58.3%) over avoiding (20.8%), and women preferred avoiding (44.2%) slightly more than compromising (43.1%) Those with a vocational secondary education (3 years) preferred compromising (66.7%), while those with a PhD mostly chose avoiding (66.7%) Longer tenure was significantly related to the predominant conflict resolution style

Patients and their family members mainly triggered WPV, and some exhibited aggressive behaviours, such as psychiatric disorders or drug influence [20, 23, 28]. Female patients in a particular age group were noted as being at risk of causing both physical and non-physical violence [34]. WPV was also prevalent in clinics, which was attributable to poor patient–professional relationships triggered by the perpetrator’s inappropriate attitude, such as being excessively demanding, or when clients did not fully understand the role of HCWs or used PHC services for malingering [25, 26, 31] (Table 3).

- Community/Geographical factors

We identified the role of the local community, where WPV was prevalent among HCWs who served at PHC facilities in drug trafficking areas [27] and that were surrounded by a population of lower socio-economic status [28]. Furthermore, WPV was increased in clinics in urban and larger districts, which have a lower HCW density per a given population compared to the national threshold of human resource requirement [29, 32, 39], whereas WPV reduced in rural areas, where medical service was perceived more accessible due to lower population density [39] (Table 3). 

- Workplace factors

The operational service, healthcare system delivery, and organisational factors were identified as the three major sub-themes of work-related predictors of WPV. Specific operational services increased the likelihood of WPV, for example, during home visit activities, handling preschool students, dealing with clients at the counter, and triaging emergency cases [27, 36–39]. WPV was more prevalent if the service was delivered by HCWs who worked extra hours with multiple shifts, particularly during the evening and night shifts [30, 36, 37, 39]. HCWs who worked in clinics with poor healthcare delivery systems due to ineffective appointment systems, uncertainty of service or waiting times, and inadequate staffing [25–27, 31, 33, 36, 37] faced higher potential exposure to aggressive events compared to those working in clinics with better systems. WPV was also linked to a lack of organisational support, mainly in fulfilling workers’ needs, such as providing sufficient human resources, capital, and on-job training, or equal pay schedule and job task distribution, or ensuring a safety climate and clear policy for WPV management [22, 26, 27, 29, 30, 33, 35–37]. We also determined that the lack of a multidisciplinary work team and devalued family medicine speciality by other specialists caused many HCWs to remain in poor intra- or inter-profession relationships and be vulnerable to co-worker-perpetrated incidents in PHC settings [24, 26, 33, 39] (Table 3).

### Effects of WPV

The most frequently reported implications by the victims of WPV involved their professional life, where most studies mentioned reduced performance, absenteeism, the decision to change practice, and feeling dissatisfied or overlooked in their roles. This was followed by poor psychological well-being (anxiety, stress, or burnout), and emotional effects (feeling guilty, ashamed, and punished) [13, 21, 24, 30, 31, 34, 35, 38]. Three studies reported on physical injuries [13, 21, 34], while only one study reported a deficit in victims’ cognitive function, which might lead to near-miss events involving patients’ safety elements, and social function defects, where some victims refused to deal with patients in the future [31]. Only one study reported the WPV implication of being environmentally damaged [34] (Table 3).

### Victims’ coping mechanisms and organisational interventions

The coping strategies adopted by HCWs varied depending on the timing of the violent events. Safety approaches such as carrying a personal alarm, bearing a chevron, and other similar steps were used, especially by female HCWs, as a proactive coping measure against potentially hazardous incidents [21]. “During an aggressive situation triggered by patients, certain workers used non-technical skills, which included leadership, task management, situational awareness, and decision-making [31]. During inter-professional conflict (physician–nurse conflict), the most predominant conflict resolution styles were compromise and avoiding, followed by accommodating, collaborating, and competing [40]. Avoiding conflict resolution was most common among nurses, whereas compromise was most common among doctors [40]. Post-violent event, most HCWs chose to take no action, while some utilised a formal reporting channel either via their supervisors, higher managers, police officers, or legal prosecution. Some HCWs also utilised informal channels by sharing problems with their social network members, such as colleagues, friends, or family members [13, 30, 36, 39]. Only one article mentioned health managers’ organisational preventive interventions, which included internal workplace rotation, staff replacement, and writing formal explanation letters [34] (Table 3).

## Discussion

We analysed the global prevalence and other vital information on WPV against HCWs who serve in the PHC setting. We identified noteworthy findings not reported in earlier systematic reviews and meta-analyses, where the healthcare setting type was not taken into primary consideration [2, 41–49].

Determining a definite judgement on WPV incidence against PHC workers worldwide is challenging, given that several of the studies selected for analysis were conducted using convenience sampling with low response rates. Nevertheless, notable results were obtained. WPV prevalence varied significantly, where the highest prevalence was reported in Germany (91%) and the lowest was reported in China (14%). Based on the average 1-year prevalence rate of WPV, we determined that the European and American regions had a greater WPV prevalence than others, which was consistent with a recent meta-analysis [50]. One reason might be the more effective reporting system in these regions, which facilitate more reports through a formal channel, as mentioned previously [51]. Contrastingly, opposite circumstances might cause WPV events to go unreported in other parts of the world. We also revealed a need for more evidence on WPV in the PHC context in Southeast–East Asia and African regions. The number of peer-reviewed articles from these regions could have been much higher, which inferred that the issue in these continents still requires resolution.

Various incidents of violence, including those of a criminal or domestic nature, commonly occur in the tertiary setting. The Healthcare Crime Survey by the International Association for Healthcare Security and Safety (IAHSS) reported that within a 10-year period (2010–2022), the number of hospital workers who experienced ten types of crime-related events in the workplace, such as murder, rape, robbery, burglary, theft (Type I), increased by the year [52]. In contrast, most studies conducted in PHC settings focused on providing more evidence of Type II violence, whereby other types (I and IV) were rarely detected. The scarcity of evidence does not necessarily indicate that PHC workers are not vulnerable to criminal or domestic violence. Rather, it implies that WPV is still not entirely explored in the PHC setting, which undermines the establishment of a comprehensive violence prevention strategy that encompasses all types of violence [53].

Hospital-based studies reported diverse forms of violence, where both physical and verbal violence were dominant [47, 54–56]. Violence as a whole and physical violence in particular tend to occur in nursing homes and certain hospital departments, such as the psychiatric department, emergency rooms, and geriatric nursing units [47, 55, 56]. Volatile individuals with serious medical conditions or psychiatric issues or who are under the influence of drugs or alcohol were mainly responsible for this severe physical aggression [53]. Similar to previous hospital-based studies, diverse forms of violence (verbal abuse, physical attacks, bullying, sexual-based violence, psychological abuse) were recorded in PHC settings. Despite this, most of the studies determined that the perpetrators’ disparate characteristics resulted in more frequent documentation of verbal violence than physical violence. Dissatisfied patients or family members were more likely to perpetrate greater incidents of verbal abuse [25, 26, 31], either due to their medical conditions or dissatisfaction with the services provided [30]. This noteworthy discovery prompted new ideas, indicating that variance in the form of violence might also be determined by the healthcare setting role [57].

Our findings demonstrated that sexual-based violence was the least frequently documented form of violence, with a regional differences pattern indicating relatively lower sexual-based violence reporting in the Middle Eastern region [13, 30]. This result contrasted with a previous systematic review of African countries that reported that sexual-based violence was one of the dominant forms of WPV. This lower incidence was possibly due to under-reporting by female employees who were reluctant to report sexual harassment aggravated by cultural sensitivities regarding sexual assault exposure [58]. Such culturally driven decision-making practices are worrying, as they could lead to underestimation of the true extent of the issues and cause more humiliating incidents and the lack of a proper response.

We identified considerable numbers of significant predisposing factors, which were determined via advanced multivariate modelling. Most factors were comparable with that in previous WPV research, especially those related to the victims’ individual socio-demographic and professional backgrounds [2, 41, 42]. Several studies consistently reported that nurses were vulnerable to WPV compared to physicians and others, which was supported by numerous prior systematic studies [19, 23, 27, 37]. This could be explained by the accessible nature of nurses as healthcare professionals to patients and families [50]. Furthermore, nurses interact first-hand with clients during treatment, rendering them more likely to become the initial victims of WPV before others. Nevertheless, this result should not necessarily suggest that other professions are not at risk for violence. Due to the shortage of evidence regarding the remaining category of PHC workers, it is impossible to provide a more conclusive and realistic assessment of the above.

The results demonstrated that many PHC clinics were built in community areas with a variety of settings, such as high-density commercial developments in urban or rural areas, resource-limited locations, or areas with a high crime concentration [27–29, 32, 39]. Therefore, an additional new sub-theme under predisposing factors, namely, “community and geographical factors”, was created to include all evidence on the relationship between WPV vulnerability and community social character and geo-spatial factors. Although several hospital-based studies deemed this topic less significant, several studies in the present review that examined the relationship between geographic information and the surrounding population characteristics with WPV reported valuable and constructive information for PHC prevention framework efforts.

In general, we identified a similar correlation between work-related factors and WPV as in hospital-based studies, particularly on healthcare system delivery and organisational support elements [40–48]. Nonetheless, the evidence on operational service was vastly distinct. As several PHC services are expanded outside facilities, there is increased potential for violence against HCWs when they provide out of clinic services, for example, during home visits and school health services [21, 37, 39]. Such situations might require more comprehensive prevention measures compared to violent events that occur within health facilities. Unfortunately, the available literature that describes and assesses the safety elements of HCWs in PHC settings mainly focused on services inside the health facilities, indicating that WPV prevention and management should be expanded to outdoor services [21].

The studies included in this review comprehensively described the observed implications on WPV victims in PHC settings. Nonetheless, additional vital information on the adverse effects on organisational elements remains lacking, especially regarding the quality of patient care involving potential near-miss events, negligence, and reduced safety elements [31]. The economic effect is another important aspect that requires further consideration. Recent financial expense data were only available from hospital-based research. A systematic review revealed that WPV events resulting in 3757 days of absence at one hospital over 1–3 years involved a cost exceeding USD 1.3 billion that was mainly due to reduced productivity [43].

The magnitude of under-reporting among HCWs was concerning, as most respondents admitted that they declined to report WPV cases through formal reporting channels, such as via electronic notification systems, supervisors, or police officers [13, 30, 36, 39]. Although the included articles mentioned several impediments to reporting, such as fear of retaliation, fear of missing one’s job, and feelings of regret and humiliation, [13, 30, 36], the main reason for under-reporting was a lack of trust in existing WPV preventive institutional policies. Most respondents perceived that reporting the case would not lead to positive changes and were dissatisfied with how the policy was administered [13, 30]. Despite much evidence on proactive coping mechanisms utilised by the HCWs, which were either behaviour change technique or conflict resolution style, we did not obtain additional crucial information on existing regional WPV policies or specific intervention frameworks at institutional level [31, 40]. Furthermore, reports of the mediating functions of federal- or state-level central funding and legal acts or regulatory support in establishing effective regional violence policies were also absent in primary settings. Further discussion in this area is crucial as significant federal or state government support would improve HCWs’ perceptions of regional prevention program and would potentially reduce the rate of violence against HCWs.

### Opportunities for future research

Only a few studies discussing WPV in the PHC setting have been published over the 10 years covered in this review. Local researchers and stakeholders should define and prioritise important areas of study. Given the heterogeneity of the forms of violence, it might be advantageous to conduct additional observational research in the future to describe the situation and investigate the associations between the rate of violence and its multiple predictors using Poisson regression analysis [59]. At the present stage, quasi-experimental evidence is ambitious. Therefore, more longitudinal studies are required to evaluate the efficacy of any newly introduced violence prevention and management measures designed in primary healthcare settings [60].

A comprehensive investigation of WPV occurrences beyond Type II violence is required to accurately reflect the breadth of the issue and focus on prevention efforts. In the present study, the association pattern between the consequences of WPV for specific perpetrators was not investigated as in prior research due to the scarcity of evidence on Type I, III, and IV violence. For example, Nowrouzi-Kia et al. revealed that the victims of inter-professional perpetuation (Type III) experienced more severe consequences involving their professional life (low job satisfaction, increased intention to quit) than those who experienced patient or family-perpetrated violence (Type II), which involved psychological and emotional changes [61, 62]. In addition, the study scope must also be expanded to include assaults against both healthcare personnel and patients in primary settings. A hospital-based investigation by Staggs 2015 revealed a significant association between the number of staff at psychiatric patient units and the frequency of violent incidents. Surprisingly, this rigorous investigation determined that higher levels of hospital staffing of registered nurses were associated with a higher assault rate against hospital staff and a lower assault rate against patients [63].

Despite universal exposure to WPV, the incidence rates and types of violence vary between regions. Thus, the primary investigation focus should be tailored to specific violence issues in a particular setting. Our results highlighted the need for further research into strengthening WPV policy, particularly concerning the reporting systems in regions outside European and American countries. Compared to other regions, local academicians in Southeast Asia and Africa are encouraged to increase their efforts to perform more epidemiological WPV studies in the future to better understand the WPV issue. It is crucial to identify the underlying causes of low prevalence of sexual harassment, particularly in the Middle East, which might be caused by under-reporting influenced by culture or gender bias. Although it is asserted that sexual-based violence is likely to occur commonly in cultures that foster beliefs of perceived male superiority and female social and cultural inferiority, the reported prevalence rate of such violence in certain regions [64], particularly in the Middle East, was low, possibly due to under-reporting. Thus, to address this persistent problem, the existing reporting mechanisms must be improved and sexual-based violence should be distinguished from other forms of violence to encourage more case reporting. Simultaneously, sexual-based violence should also be defined differently across countries and various social and cultural contexts to reduce impediments to reporting [64].

In existing studies, the main focus of work-related predisposing factors is based on superficial situational analysis, which is identified using the local version of the standard WPV instrument tool via a quantitative approach. Nevertheless, this weak evidence would not support a more effective preventive WPV framework. This issue should be addressed in more depth and involve psychosocial workplace elements that cover interpersonal interactions at work and individual work and its effects on employees, organisational conditions, and culture. Qualitative investigations that complement and contextualise quantitative findings is one means of obtaining a greater understanding and more viewpoints.

### Implications of WPV policies

The results had major effects on WPV prevention and intervention policies in the PHC setting. The results highlighted the importance of enacting supportive organisational conditions, such as providing adequate staffing, adjusting working hours to acceptable shifts, or developing education and training programmes. As part of a holistic solution to violence, training programmes should focus on recognising early indicators of possible violence, assertiveness approaches, redirection strategies, and patient management protocols to mitigate negative effects on physical, psychological, and professional well-being. While previous WPV studies focused more on physical violence and inspired intervention efforts in many organisational settings, our results necessitate attention on non-physical forms of violence, which include verbal harassment, sexual misconduct, and intimidation. The increased potential of domestic- and crime-type violence in PHC settings necessitates expanded prevention programmes that address patients, visitors, healthcare providers, the surrounding community, and the general population.

Our results demonstrated that under-reporting of violent events remains a key issue, which is attributable to a lack of standardised WPV policies in many PHC settings. The initial action that should be implemented in accordance with human resource policy is to establish a system that renders it mandatory for victims, witnesses, and supervisors to report known instances of violence to HCWs. Unnecessary and redundant reporting processes can be reduced by an advanced system for rapidly recording WPV incidents, such as in hospital settings, where WPV is reported via a centralised electronic system. However, healthcare professional and organisational advocacy remains necessary. These parties must promote the value of routine procedures to ask employees about their encounters with patient violence and to foster an environment, where the organisation encourages reporting of violent incidents.

In addition to insufficient reporting, it is crucial to draw attention to the manner in which violent incident investigations are currently conducted in most workplaces. In reality, the incident reporting focuses on the violence itself and its superficial or circumstantial analysis, as opposed to an in-depth examination of the causes of violence, which are due to workplace psychosocial hazards, poor clinic environment, or poor customer service. For example, if any patient-inflicted violence occurred as a result of unsatisfactory conditions caused by poor clinic service, such as unnecessary delay, the tendency is to report on the perpetrator’s behaviour or on the violence itself rather than the unmet health service provision issue. In the long-term, however, the findings of such an investigation would not support the development of a violence prevention and management guideline, as it focuses on addressing aggressive patients rather than enhancing clinic service quality. Therefore, the relevant authorities should formulate a proper plan to improve the existing reporting and investigations mechanism to ensure that it is more comprehensive, structured, and detailed, either by providing proper training for the investigators or conducting institutional-level routine root cause analysis discussions, so that the violence hazard risk assessment can be framed effectively to resolve the antecedent factors in the future.

Nonetheless, there remains much room for primary-level improvement in WPV awareness and abilities. Reports on the mediating roles of federal- and state-level central funding and regulatory support for efficient local WPV policies at primary level have not been found. Therefore, more studies will be necessary to fill these gaps and concentrate on examining the relationship between regional WPV policies and national support. Possibly, more central funding and state regulation following new positive results can be made available to aid local preventive programs. A strong central financial support is essential to support regional preventive programmes, such as employing security guards, enhancing the physical security of health facilities buildings, and research grants. Awadalla and Roughton strongly suggested that adequate national-level financial support is one of the essential components of successful regional policies that would alter HCW perceptions [65]. In terms of law and regulation, for example, Ferris and Murphy firmly supported the role of the Occupational Safety and Health Act (OSHA) via the issuance of the “Enforcement Procedures for Investigating or Inspecting Workplace Violence” instructions to institutional-level officers as one of the essential components of local WPV prevention strategies [66].

### Study strength and limitations

The present study is a preliminary systematic review that explored evidence of WPV against all PHC workers in empirical studies worldwide. The breadth of the review was achieved by incorporating numerous peer-reviewed high-quality published studies, which enabled us to derive a solid conclusion. The approach relied on the authors’ prior knowledge of the study topic, the standard review technique, and specialised keywords.

It is also important to emphasise several potential limitations. First, recall bias was introduced in most studies as the authors used self-reporting to recall previous incidents either up to 12 months prior or after a lifetime. As most of the included studies involved small sample sizes, a few studies with low response rates restricted the generalisability of the findings. Several studies were descriptive and were cross-sectional; consequently, extra caution should be applied when making inferences pertaining to the risk factor interactions with violence. Variability in the instrument used, data collection and analysis methods, the notion of violence, and the general study objective might account for the heterogeneity across studies, which limited comparisons across studies. As PHC health system delivery between countries is described by different terms or names or might be identified by names besides those used in the present study, studies that use such terms might have been overlooked during the database search.

## Conclusion

WPV in the PHC setting is a common and growing issue worldwide. Many PHC workers reported experiencing violence in recent years, strongly suggesting that violence is a well-recognised psychosocial hazard in PHC comparable to hospital settings. HCWs are highly susceptible to violence perpetrated by patients or their families, which results in considerable negative consequences. With various predisposing factors, this complex issue indicates a need for more serious consideration of a resolution on par with that in the tertiary setting. Several research gaps and limitations necessitate additional rigorous analytical and interventional research in the future. Information on violent events must be comprehensively collected to delineate the complete scope of the issue and formulate prevention strategies based on potentially modifiable risk factors. Thus, a new interventions framework to mitigate violent events and control their negative implications can be established. The results presented here were derived from literature on diverse cultures worldwide, and, therefore, can be used as a data reference for policymakers and academicians for future opportunities in the healthcare system field.

## Data Availability

All data generated or analysed during this study are included in this published article.
